# Increased Anti-Inflammatory Therapeutic Potential and Progenitor Marker Expression of Corneal Mesenchymal Stem Cells Cultured in an Optimized Propagation Medium

**DOI:** 10.1177/09636897241241992

**Published:** 2024-04-11

**Authors:** Andrew Hopkinson, Maria Notara, Claus Cursiefen, Laura E. Sidney

**Affiliations:** 1 Academic Ophthalmology, Mental Health and Clinical Neurosciences, University of Nottingham, Nottingham, UK; 2 Department of Ophthalmology, Faculty of Medicine and University Hospital Cologne, University of Cologne, Koln, Germany; 3 Regenerating and Modelling Tissues, Translational Medical Sciences, School of Medicine, University of Nottingham, Nottingham, UK

**Keywords:** cornea, mesenchymal stem cells, CD34, cell therapy, dry eye, inflammation

## Abstract

There is a huge unmet need for new treatment modalities for ocular surface inflammatory disorders (OSIDs) such as dry eye disease and meibomian gland dysfunction. Mesenchymal stem cell therapies may hold the answer due to their potent immunomodulatory properties, low immunogenicity, and ability to modulate both the innate and adaptive immune response. MSC-like cells that can be isolated from the corneal stroma (C-MSCs) offer a potential new treatment strategy; however, an optimized culture medium needs to be developed to produce the ideal phenotype for use in a cell therapy to treat OSIDs. The effects of *in vitro* expansion of human C-MSC in a medium of M199 containing fetal bovine serum (FBS) was compared to a stem cell medium (SCM) containing knockout serum replacement (KSR) with basic fibroblast growth factor (bFGF) and human leukemia inhibitory factor (LIF), investigating viability, protein, and gene expression. Isolating populations expressing CD34 or using siRNA knockdown of CD34 were investigated. Finally, the potential of C-MSC as a cell therapy was assessed using co-culture with an *in vitro* corneal epithelial cell injury model and the angiogenic effects of C-MSC conditioned medium were evaluated with blood and lymph endothelial cells. Both media supported proliferation of C-MSC, with SCM increasing expression of *CD34*, *ABCG2*, *PAX6, NANOG, REX1, SOX2*, and *THY1*, supported by increased associated protein expression. Isolating cell populations expressing CD34 protein made little difference to gene expression, however, knockdown of the *CD34* gene led to decreased expression of progenitor genes. C-MSC increased viability of injured corneal epithelial cells whilst decreasing levels of cytotoxicity and interleukins-6 and -8. No pro-angiogenic effect of C-MSC was seen. Culture medium can significantly influence C-MSC phenotype and culture in SCM produced a cell phenotype more suitable for further consideration as an anti-inflammatory cell therapy. C-MSC show considerable potential for development as therapies for OSIDs, acting through anti-inflammatory action.

## Introduction

Treatment of corneal diseases poses a unique therapeutic challenge due to its specialized cellular and structural organization, essential to maintain transparency required for effective vision. Ocular surface inflammatory disorders (OSIDs) occur when the tightly regulated homeostasis at the ocular surface is disturbed, and encompass a range of heterogeneous diseases with a variety of etiologies and symptoms, where inflammation plays a critical role in pathogenesis^1^. Cicatricial conjunctivitis, Steven’s Johnson Syndrome, dry eye disease, meibomian gland dysfunction, allergic eye diseases, chemical eye burn, trauma, iatrogenic insult following corneal and/or refractive surgery, and contact lens-related complications are the common examples of OSIDs that are frequently encountered and managed in clinical practice^2^.

Due to the abundance of therapeutic factors produced by human stem cells, regenerative medicine may hold the key to developing a superior treatment to alleviate OSIDs with associated limbal stem cell deficiency. Mesenchymal stem cell (MSC)-based therapies are at the forefront of regenerative medicine due to their potent immunomodulatory properties, relatively low immunogenicity, and ability to modulate both the innate and adaptive immune response^3,4^. The discovery and characterization of an MSC-like cell that can be derived from stromal cells found in the limbal region of the cornea offers a potential new treatment pathway for OSIDs, and an alternative to the more commonly investigated bone marrow or adipose-derived MSC^5–8^.

MSCs can be isolated from most tissues in the body and cultured *in vitro*, however they do not all possess the same properties. For example, literature demonstrating MSC secretion of the anti-inflammatory cytokine, IL-10, is highly contradictory, and could be due to the source of the cells^9^. Each tissue-specific MSC niche is different, leading to a risk of cells exhibiting unexpected behavior when transplanted into a separate tissue. Therefore, there may be therapeutic benefits to transplanting tissue-specific MSCs already conditioned to the corneal microenvironment, back onto the ocular surface. It has been demonstrated that when isolated and expanded *in vitro*, keratocytes from the corneal limbal stroma assume an MSC phenotype^5,7,10–12^. Furthermore, these corneal MSCs (C-MSCs) show anti-inflammatory potential when co-cultured with injured corneal epithelial cells^6^, can reduce corneal scarring after wounding^13^, and express specific markers of the cornea when other MSC types do not^7,14^.

Culture of cells extracted from the corneal stroma has been performed using many different media and conditions, with the intention of either retaining keratocyte phenotype or promoting a stem cell phenotype. Traditionally, a medium of Dulbecco’s modified Eagle’s medium (DMEM) supplemented with 10% fetal bovine serum (FBS) has been widely used^15–17^; however, this has been shown to produce sub-optimal culture conditions for the production of MSC^12,18^. Culture in a medium containing serum, of varying concentrations, is usually preferred, as it provides a source of attachment and growth factors, allowing proliferation and rapid expansion of the cells. The use of M199 basal medium with the addition of 20% FBS generates MSC that adhere to International Society of Cellular Therapy (ISCT) criteria^11^. However, serum, or more specifically growth factors present in serum, such as transforming growth factor-β1, has also been reported to cause fibroblastic or myofibroblastic differentiation, characterized by a fusiform morphology and protein markers such as α-SMA^19,20^ and CD90^21^. Use of medium containing a knockout serum replacement (KSR) also produces a phenotype similar to that stated in the ISCT criteria but with reduced production of myofibroblastic cells^12^. In addition, including factors such a basic fibroblast growth factor (bFGF) and human leukemia inhibitory factor (LIF) have also been shown to induce a phenotype indicative of a pluripotent stem cell^22–24^.

In this study, we aimed to perform an in-depth investigation of C-MSC characteristics, comparing cells cultured in a basic medium of M199 media containing FBS (referred to throughout as M199) to those cultured in media containing KSR (referred to throughout as SCM, Stem Cell Medium). From our previous study, which encompassed a wider range of media^12^, the number of markers compared have been increased, investigating both traditionally associated MSC markers (CD73, CD90 and CD105), and markers associated with progenitor cells and pluripotent cells (CD34, ABCG2, Oct4, Nanog, Rex1 and Sox2). Within this study, we have also aimed to extend our investigations into the progenitor marker CD34 and the effect that expression of this marker has on the overall population of cells, elucidating whether this is also affected by culture medium choice. Lastly, the ability of C-MSC cultured in both media to produce an anti-inflammatory effect on injured corneal epithelial cells was explored, assessing the pro- or anti-angiogenic role of C-MSC when used to stimulate blood and lymphatic endothelial cells. This characterization was performed with the intent of producing C-MSC with a phenotype optimally suited to treating OSIDs as a topical therapy for the front of the eye.

## Materials and Methods

### Materials

Reagents were purchased from Thermo Fisher Scientific, UK, unless otherwise stated.

### Tissue

Anonymized human corneas for research were obtained from Manchester Eye Bank (NHS Blood and Transplant) subject to a Materials Transfer Agreement. All research, storage and disposal of human tissue was performed under a research license from the Human Tissue Authority.

### Isolation and Culture of Primary Human Corneal Stromal Cells

Human C-MSC were isolated from corneoscleral rims using collagenase digestion, as previously described^5^. Primary C-MSC were continually cultured in either M199 medium consisting of M199 (Sigma-Aldrich, Poole, UK) with 20% (v/v) FBS (Sigma-Aldrich), 2 mM L-glutamine and antibiotics (20 ng/mL gentamicin, 0.5 ng/mL amphotericin B); or Stem Cell Medium (SCM) consisting of DMEM/F12 with Glutamax supplemented with 20% (v/v) knockout serum replacement (KSR), 1% (v/v) non-essential amino acids, 4 ng/mL bFGF, 5 ng/mL hLIF (New England Biolabs, Hertfordshire, UK) and antibiotics. C-MSCs in both media were cultured on surfaces coated with 0.1% (v/v) bovine gelatin (Sigma-Aldrich). Cells were passaged using treatment with TrypLE™ Express and seeded for both passage and experiment at 30,000 cells/cm^2^. At least three different donors of human C-MSC, with at least three experimental repeats for each donor were used in each experiment. Specific information on n-number for individual experiments is given in the figure legend.

### Immunocytochemistry

Cell samples for immunocytochemistry were cultured in glass chamber slides (Nunc Lab-Tek, Thermo Fisher Scientific, UK) and immunocytochemistry was performed as described previously^5,12^. See Supplemental Table 1 for primary antibody details. Secondary antibodies were donkey Alexa Fluor-488, -546 or -594. Counterstaining of actin filaments with Alexa Fluor-488 conjugated phalloidin (dilution 1:40), and nuclei with 0.5 μg/mL 4′,6-diamidino-2-phenylindole (DAPI; Santa Cruz Biotechnology, Heidelberg, Germany), was additionally performed in some cases. Samples were imaged using an upright fluorescence microscope (BX51, Olympus), a black and white camera (XM-10, Olympus) and Cell^F software (Olympus). Phase contrast imaging was performed on a Leica DM-IRB inverted microscope, and images captured with a Hammatsu digital camera and Volocity imaging software (Improvision, Coventry, UK).

### C-MSC Proliferation and Viability

Cell proliferation and viability was assessed in all media using PrestoBlue^™^ Cell Viability Reagent (Life Technologies), as described previously^12^. Briefly, cells were washed in PBS and 10% (v/v) PrestoBlue reagent added to each well and incubated for 20 min at 37°C. Fluorescence readings were taken at excitation 560 nm/emission 590 nm using a CLARIOstar plate reader (BMG LABTECH, Buckinghamshire, UK).

### Quantitative Reverse Transcription Polymerase Chain Reaction (RT-qPCR)

RT-qPCR was performed as described previously^14^. RNA was extracted using an RNeasy mini kit (Qiagen, Manchester, UK), and 1 μg of RNA was transcribed into single stranded cDNA using Superscript III reverse transcriptase. For PCR reactions, 1 μL of cDNA was used with inventoried Taqman assays to detect genes of interest (see Supplemental Table 2). Amplification was performed on an Mx3005P qPCR system (Stratagene, Agilent Technologies, UK). Reactions were analyzed using the Real Time PCR Miner algorithm^25^ and all values were normalized to readings of the endogenous reference gene glyceraldehyde 3-phosphate dehydrogenase (*GAPDH*).

### Flow Cytometry

C-MSC were detached from the flask and the cell suspension diluted in ice cold flow buffer, consisting of PBS with 10% (v/v) FBS and 0.1% (w/v) sodium azide. For cell surface staining, 95 µL of cell suspension was added to individual flow tubes with 5 µL of conjugated antibody (see Supplementary Table 3) and incubated at 4^o^C for 30 min. In addition, cells were identically prepared containing no stain, or 5 µL of FITC, PE or PE-Cy5 isotype for controls. Following incubation, 1 mL PBS buffer was added to each sample, and the cells were centrifuged at 250 × *g* for 5 min. Supernatant was aspirated, and following another wash in 1 mL PBS buffer, cells were resuspended in 400 µL 4% (v/v) paraformaldehyde (PFA). Flow cytometry was performed using the BD FACSCanto II and Beckman Coulter Kaluza Analysis Software was utilized for cell gating and analysis.

### Magnetic-Activated Cell Sorting (MACS)

C-MSC cell suspensions were incubated with 5 μL biotin-conjugated mouse monoclonal antibody to human CD34 (clone 581, Thermo Fisher Scientific) for 30 min at room temperature. Subsequently, cells were incubated with anti-biotin magnetic microbeads (Miltenyi Biotec) for 15 min at 4°C. Cells were sorted for CD34 expression using the MiniMACS™ separator in combination with MS columns (Miltenyi).

### siRNA-Mediated Knockdown of CD34

Human CD34 siRNA (SMARTpool: ON-TARGETplus; L-019503-00-005, sequences 5′-UAACCUCAGUUUAUGGAAA−3′, 5′-GCACUAGCCUUGCAACAUCC−3′, 5′-GCGCUUUGCUUGCUGAGUU−3′, and 5′-CCACUAAACCCUAUACAUC−3′) and non-targeting siRNA (ON-TARGETplus #1) were obtained from Dharmacon, GE Lifesciences, Buckinghamshire, UK. For transfection, C-MSC were seeded at 3.2 × 10^3^ cells/cm^2^ and transfection was performed using the N-TER nanoparticle siRNA transfection system (Sigma-Aldrich), according to manufacturer’s instructions. For effective knockdown of CD34, transfection was performed twice on the same cells, with the second transfection occurring 48 h after the first. Viability measurements were taken 48 h after each transfection. RNA was collected for RT-qPCR analysis, 48 h after the second transfection.

### LPS Stimulation of C-MSC

C-MSC at P4 were plated at 1.05 × 10^4^ cells/cm^2^ in 12-well plates and cultured overnight before incubation in medium containing 1 µg/mL lipopolysaccharide (LPS, Sigma-Aldrich), from *Escherichia coli.* After incubation for 72 h, the supernatant was collected, and cell viability assessed.

### Corneal Epithelial Cell Injury Model

SV40-immortalized human corneal epithelial cells (ihCEC)^26^ were cultured in supplemented EpiLife containing 5 mL human keratinocyte growth supplement and antibiotics as previously described^6^. For the injury model, ihCEC were seeded at 2.6 × 10^4^ cells/cm^2^ in 12-well plates and cultured for 72 h. Injury was induced by treating cells with 20% (v/v) ethanol in PBS for 30 s, washing three times in PBS to remove alcohol remnants, before stimulation in 1 µg/mL LPS in EpiLife for 72 h. For co-cultures, C-MSC were seeded into transwells and transferred to ihCEC plates, after ethanol treatment. Co-cultures were then stimulated simultaneously with LPS in EpiLife for 72 h. Non-injured controls and ihCEC/C-MSC only controls were performed in parallel.

### Cytotoxicity, Nitrite, IL-6 and IL-8 Production

Cytotoxicity was assessed using Pierce™ Lactate Dehydrogenase (LDH) assay (Thermo Fisher Scientific) according to manufacturer’s instructions. Absorbance was read at 490 nm and cytotoxicity was calculated using a maximum LDH released control.

Nitrite in the culture medium was measured as an estimate of nitric oxide (NO) production, using the Griess Reagent System (Promega, Southampton, UK) according to manufacturer’s instructions. Absorbance was read at 540 nm, and nitrite concentration calculated via standard curve.

IL-6 and IL-8 within culture supernatants were assessed using enzyme-linked immunosorbent assays (ELISA, Quantikine, R&D Systems, Bio-Techne, Oxfordshire, UK), according to manufacturer’s instructions. Samples were diluted 5-fold before assaying. Optical absorbance was read at 450 nm, with correction at 540 nm and concentrations were calculated using a known standard.

### Culture of Lymphatic and Blood Endothelial Cells

Primary lymphatic and blood endothelial cells (LEC and BEC, respectively) were purchased from PromoCell and maintained in supplemented ECGM MV2 (endothelial cell growth medium) culture medium according to manufacturer’s instructions. Briefly, the cells were passaged when approximately 80% confluent by treatment with a Trypsin/EDTA (0.04%/0.03%) solution for 2 min followed by 0.05% Trypsin Inhibitor in 0.1% BSA, both reagents by PromoCell. The cells were only used until the 8th passage.

### Collection of C-MSC-Conditioned Medium

C-MSC at P4 were seeded at 10,000 cells/cm^2^ in T75 cm^2^ and 10 mL serum-free DMEM/F12 added. After 48 h conditioned medium (CM) was collected and filtered (0.2 µm filter). Prior to use in angiogenesis experiments, FBS was added at 1% (v/v).

### LEC and BEC Metabolic Activity and Proliferation

Alamar blue assays were performed according to manufacturer’s instructions. Cells were seeded in 96-well plates at a density of 5000 cells/well and left overnight in ECGM to attach. Medium was changed to CM from M199-C-MSC and SCM-C-MSC, alongside controls of DMEM/F12 with 1% FBS (non-conditioned medium) and ECGM. After 24 h, cultures were incubated with 10% (v/v) Alamar blue in PBS for 1 h and absorbance measured at 570 nm and 600 nm in an Epoch plate reader (Biotech).

### Scratch Wound Assay

Scratch wound assays were performed as described previously^27^. Briefly, LEC and BEC were seeded at 20,000 cells/well and left to adhere overnight. Monolayers were scratch-wounded using a 10 µL pipette tip and culture medium changed to CM and control medium. Images of the scratch were taken at 0 h, 2 h, 4 h, and 7 h. Scratch closure was analyzed by measuring the scratch area at each time point, using ImageJ version 1.49V, and calculating the percentage healed compared to the 0 h time point.

### Tube Formation Assay

Tube formation assays were performed as described previously^27^ on Matrigel in μ-Slide angiogenesis assay (Ibidi) slides. LEC and BEC were seeded at 5000 cells/well and incubated for 2 h for adherence, before the medium was changed to CM. Images of tube and network formation were taken after 18 h and analyzed for the number of branches, loops and branch points formed, using the lymphatic vessel analysis plugin in ImageJ version 1.49V.

### Statistical Analysis

Statistical significances were analyzed using GraphPad Prism version 6.07. Comparisons between two groups were performed using unpaired Student’s t-test and multiple groups were compared using two-way ANOVA with post-hoc Tukey’s multiple comparison test.

## Results

### Morphology and Proliferation of C-MSC in M199 and SCM

**Figure 1.**
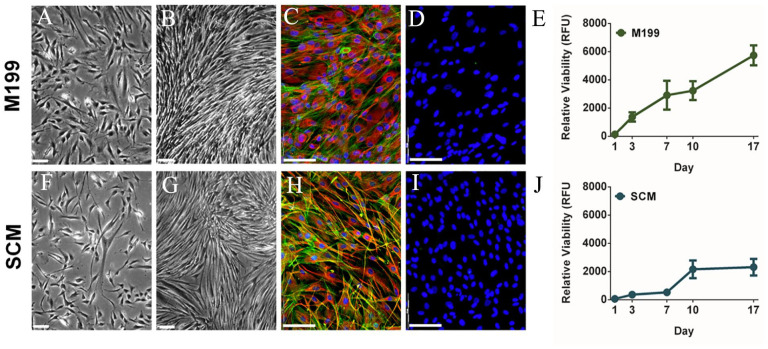
Effect of culture medium on morphology and proliferation of C-MSC. Cells were cultured in M199 (A-E) or SCM (F-J). (A, F) Representative phase contrast images at day 3 of culture (scale bar = 90 μm). (B, G) Representative phase contrast images at day 7 of culture (scale bar = 90 μm). (C, H) Representative images of vimentin expression (red) counterstained with phalloidin (F-actin, green) and DAPI (blue, scale bar=100 μm). (D, I) Representative images of Pan-Cytokeratin staining (green) counterstained with DAPI (blue, scale bar=100 μm). (E, J) Relative viability of C-MSC cultured 17 days (RFU, relative fluorescence units). Data shown as mean ± SEM of three independent experiments with different C-MSC donors (n = 3) each with six replicates.

Effects on C-MSC morphology caused by culture in M199 or SCM, were compared by imaging at 3 and 7 days culture (Fig. 1). Cell morphology initially appeared similar in both media types (Fig. 1A, F); however, C-MSC cultured in SCM appeared slightly thinner and more elongated. By day 7, cells in M199 (Fig. 1B) were very confluent and had started to spontaneously align. Cells in SCM were less confluent (Fig. 1G). Dual F-actin and vimentin staining at day 7, revealed that cells cultured in M199 (Fig. 1C) were larger, showed strong vimentin staining with filaments circling the nucleus and spreading throughout the cell. Cells in SCM (Fig. 1H) were smaller and more spindle shaped than M199, stained more intensely for actin fibers and had longer cellular processes. There was no staining for pan cytokeratin (clone C-11) in either media (Fig. 1D, I), confirming no epithelial contamination in either media. Proliferation assays supported the phase contrast imaging, showing cells in M199 (Fig. 1E) had rapid proliferation rates and cells in SCM (Fig. 1J) had a much slower proliferation rate, which increased at day 7.

### Comparative Effect of Culture Medium and Passage on Gene Expression of C-MSC

**Figure 2.**
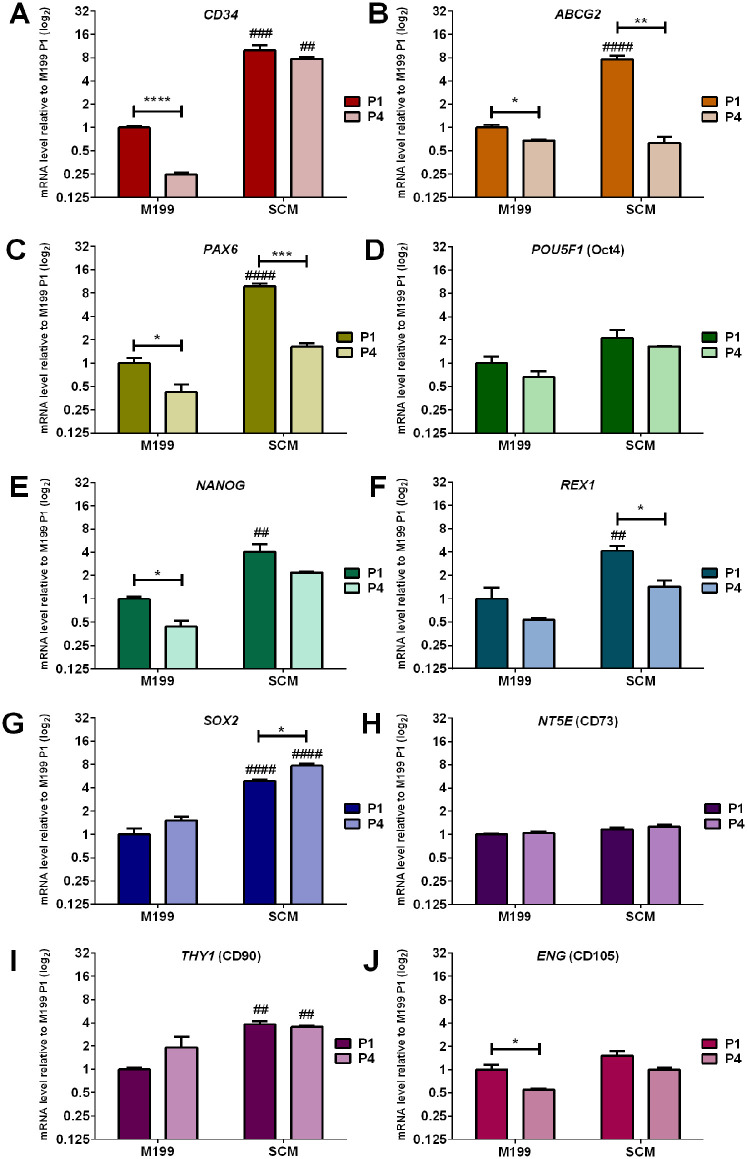
Comparative effect of culture medium on mRNA expression of C-MSC. Cells were continually cultured in either M199 or SCM. Relative levels of mRNA were determined by RT-qPCR for the following genes (A) *CD34* (B) *ABCG2*, (C) *PAX6*, (D) *POU5F1*, (E) *NANOG*, (F) *REX1*, (G) *SOX2*, (H) *NT5E*, (I) *THY1*, (J) *ENG*. C-MSC were analyzed at the end of passage 1 (P1) and passage 4 (P4). Expression of each target gene was normalized to *GAPDH* and represented relative to mRNA expression of M199 at P1. Data shown as mean ± SEM of three independent experiments with different C-MSC donors (n = 3), each with three replicates. Statistical significance of P1 vs. P4 of same medium: *P ≤ 0.05, **P ≤ 0.01, ***P ≤ 0.001. Statistical significance SCM vs. M199 P1: ##*P* ≤ 0.01, ###*P* ≤ 0.001, ####*P* ≤ 0.0001.

Changes in gene expression between C-MSC cultured in M199 and SCM at passage 1 (P1) and passage 4 (P4) was investigated by RT-qPCR analysis of genes for MSC markers (*NT5E, THY1*, *ENG*), progenitor markers (*CD34*, *ABCG2*, *PAX6*) and pluripotency markers (*POU5F1*, *NANOG*, *REX1*, *SOX2*) (Fig. 2). PCR revealed that at P1, C-MSC cultured in SCM expressed significantly more *CD34* (Fig. 2A), *ABCG2* (Fig. 2B), *PAX6* (Fig. 2C), *NANOG* (Fig. 2E), *REX1* (Fig. 2F), *SOX2* (Fig. 2G) and *THY1* (Fig. 2I), than C-MSC cultured in M199. The expression of *CD34*, *SOX2* and *THY1* was also maintained in SCM between P1 and P4. Expression of *ABCG2* and *PAX*6 was significantly decreased from P1 to P4 in both M199 and SCM. Expression of *CD34*, *NANOG*, and *ENG* (Fig. 2J) were significantly decreased when passaged in M199 but not SCM. There were no significant differences in *POU5F1* (Fig. 2D) and *NT5E* (Fig. 2H) expression due to either culture media or passage.

### Comparative Effect of Culture Medium and Passage of Protein Expression of C-MSC

**Figure 3.**
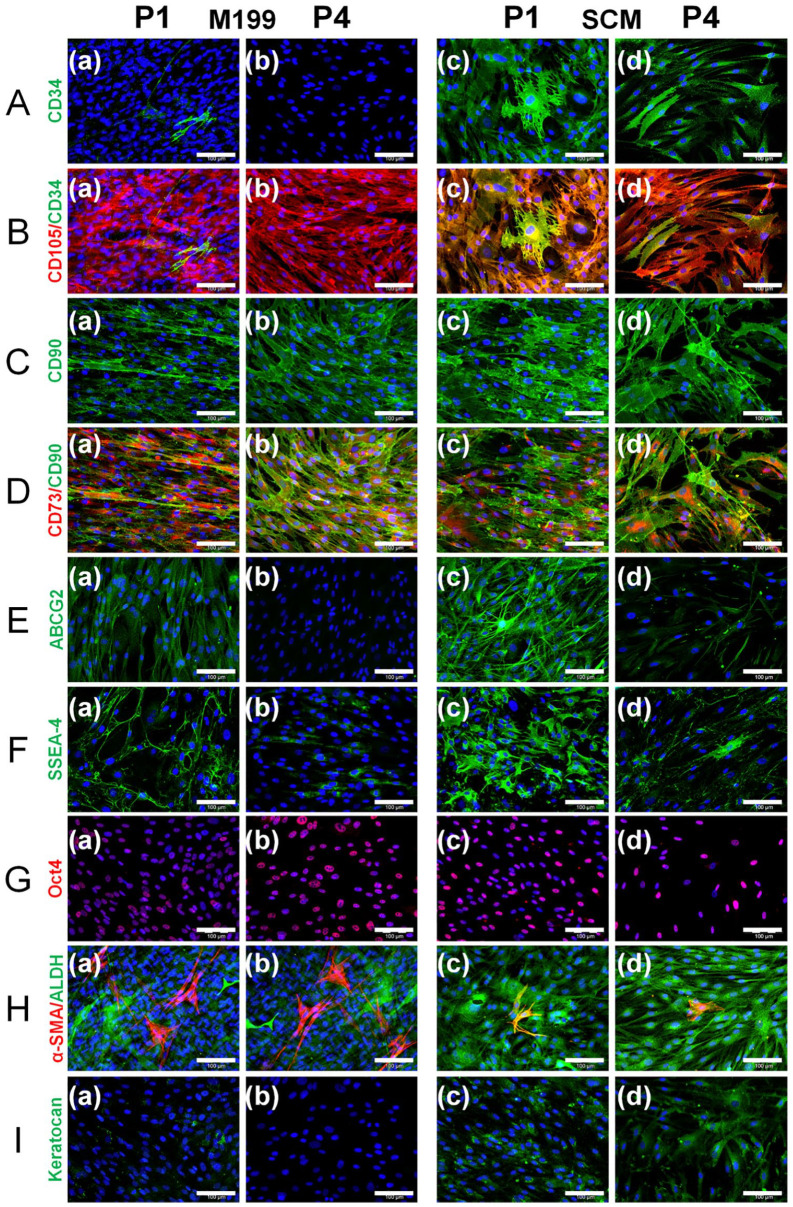
Comparative effect of culture medium on protein expression of C-MSC at P1 and P4. C-MSC were continually cultured in (a, b) M199 or (c, d) SCM and immunocytochemistry was performed at passage 1 (P1; a, c) and passage 4 (P4; b, d) for (A) CD34 (green), (B) CD105 (red) shown merged with CD34 (green), (C) CD90 (green), (D) CD73 (red) shown merged with CD90 (green), I ABCG2 (green), (F) SSEA-4 (green), (G) Oct4A (red), (H) α-SMA (red) shown merged with ALDH3A1 (green), and (I) keratocan (green). Representative images shown of three independent experiments with three different C-MSC donors (n = 3) with DAPI counterstain (blue), scale bar=100 μm.

Immunocytochemistry was performed on C-MSC after culture in M199 or SCM at to P1 and P4 (Fig. 3). At P1, staining showed that in M199, CD34 was present in a small number of individual cells (Fig. 3A). However, in SCM there was a much higher proportion of CD34 staining (Fig. 3A). At P4, there were no cells expressing CD34 in M199, but CD34-expressing cells can still be seen in SCM. CD105 (Fig. 3B), CD90 (Fig. 3C) and CD73 (Fig. 3D) were expressed by all cells in both media at both passages. ABCG2 (Fig. 3E) and SSEA-4 (Fig. 3F) stained brightly in C-MSC in SCM at P1 and was also seen in cells in M199. At P4, ABCG2 expression has disappeared in M199 but persisted in SCM. SSEA-4, although still present in both media, appears to have decreased in staining intensity. Oct4 (Fig. 3G) a transcription factor can be seen in the nucleus of cells cultured in both media at both passages. Staining for α-SMA (Fig. 3H) reveals that there is the highest proportion of myofibroblasts within M199 at both passages compared to SCM. ALDH3A1 (Fig. 3H) was subjectively appeared to stain brighter in SCM compared to M199. Finally, SCM supported production of keratocan from P1 to P4 (Fig. 3I).

**Figure 4.**
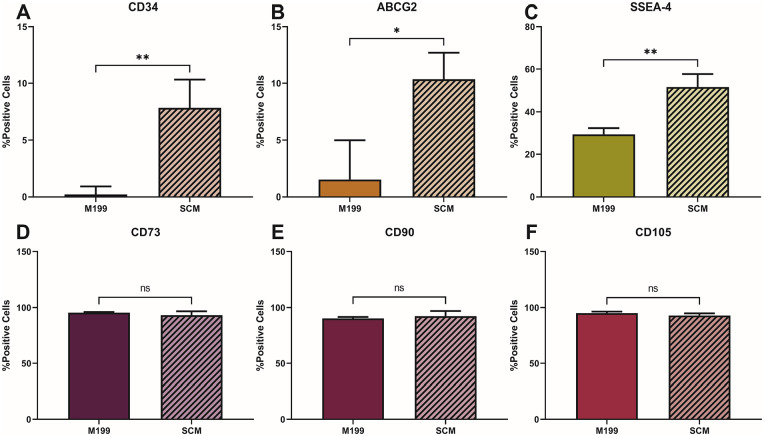
Cell-surface marker profiling of C-MSC culture in M199 and SCM at P4. C-MSC were continually cultured in M199 or SCM and flow cytometry performed at P4 for the following cell surface markers: (A) CD34, (B) ABCG2, (C) SSEA-4, (D) CD73, (E) CD90, (F) CD105). Data shown as mean ± SEM of three independent experiments with three different C-MSC donors (n = 3), each with two replicates. Statistical significance of SCM vs. M199: *P ≤ 0.05, **P ≤ 0.01.

Flow cytometry was performed on C-MSC in M199 and SCM at P4 to complement the immunocytochemistry (Fig. 4). In SCM, there was a significantly higher percentage of cells expressing CD34 (Fig. 4A), ABCG2 (Fig. 4B) and SSEA-4 (Fig. 4C) than in M199, reflecting the immunocytochemistry results. No significant difference was seen in the number of cells expressing CD73 (Fig. 4D), CD90 (Fig. 4E), and CD105 (fig. 4F) between those cultured in M199 compared to SCM.

### Differences in Gene Expression of Isolated CD34^+^ Cells

**Figure 5.**
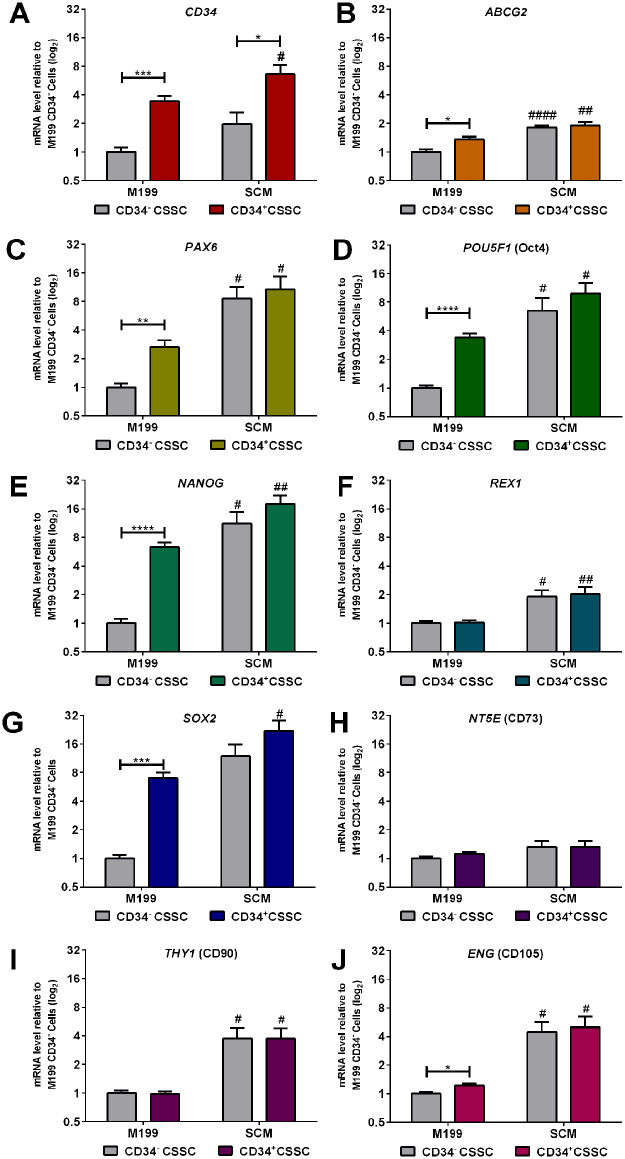
Differences in gene expression between CD34^+^ and CD34^−^ C-MSC. Cells that were cultured in either SCM or M199 were sorted at P1 for CD34 expression. Relative levels of mRNA were determined by RT-qPCR for the following genes (A) *CD34* (B) *ABCG2*, (C) *PAX6*, (D) *POU5F1*, (E) *NANOG*, (F) *REX1*, (G) *SOX2*, (H) *NT5E*, (I) *THY1*, (J) *ENG*. Expression of each target gene was normalized to *GAPDH* and represented relative to mRNA expression in CD34^−^ cells in M199. Data shown as mean ± SEM of five independent experiments with five different C-MSC donors (n = 5), each with two replicates. Statistical significance of CD34^−^ vs. CD34^+^ in M199 or SCM: *P ≤ 0.05, **P ≤ 0.01, ***P ≤ 0.001, ****P ≤ 0.0001. Statistical significance of M199 vs. SCM for CD34^−^ or CD34^+^ cells: #*P* ≤ 0.05, ##*P* ≤ 0.01, ####*P* ≤ 0.0001.

C-MSC were cultured for one passage in either SCM or M199 before sorting for positive CD34 expression by MACS. Percentage of cells expressing CD34 for each media was as follows: M199 CD34^+^ 21.13 ± 7.15; SCM CD34^+^ 33.50 ± 3.34 (see Supplementary Fig. 1). RT-qPCR was then performed to discern any differences in expression of other genes and any differences between cell populations in the two media (Fig. 5). C-MSC cultured in M199 showed more significant differences in gene expression between the CD34^+^ and CD34^−^ populations, with significantly greater expression of *CD34* (Fig. 5A), *ABCG2* (Fig. 5B), *PAX6* (Fig. 5C), *POU5F1* (Fig. 5D), *NANOG* (Fig. 5E), *SOX2* (Fig. 5G), and *ENG* (Fig. 5J) in CD34^+^ cells. C-MSC cultured in SCM demonstrated little difference in gene expression between the CD34^+^ and CD34^−^ populations with a significant difference seen only in *CD34*, as would have been expected due to sorting. In both the CD34^−^ and CD34^+^ populations, expression of all genes, with the exception of *REX1* (Fig. 5F) and *NT5E* (Fig. 5H), was significantly higher in SCM than in M199. This suggests that SCM maintains a more heterogeneous, stem cell phenotype than M199 that is not dependant on CD34 protein expression.

### Effect of siRNA-Mediated Knockdown of CD34 on C-MSC Gene Expression

**Figure 6.**
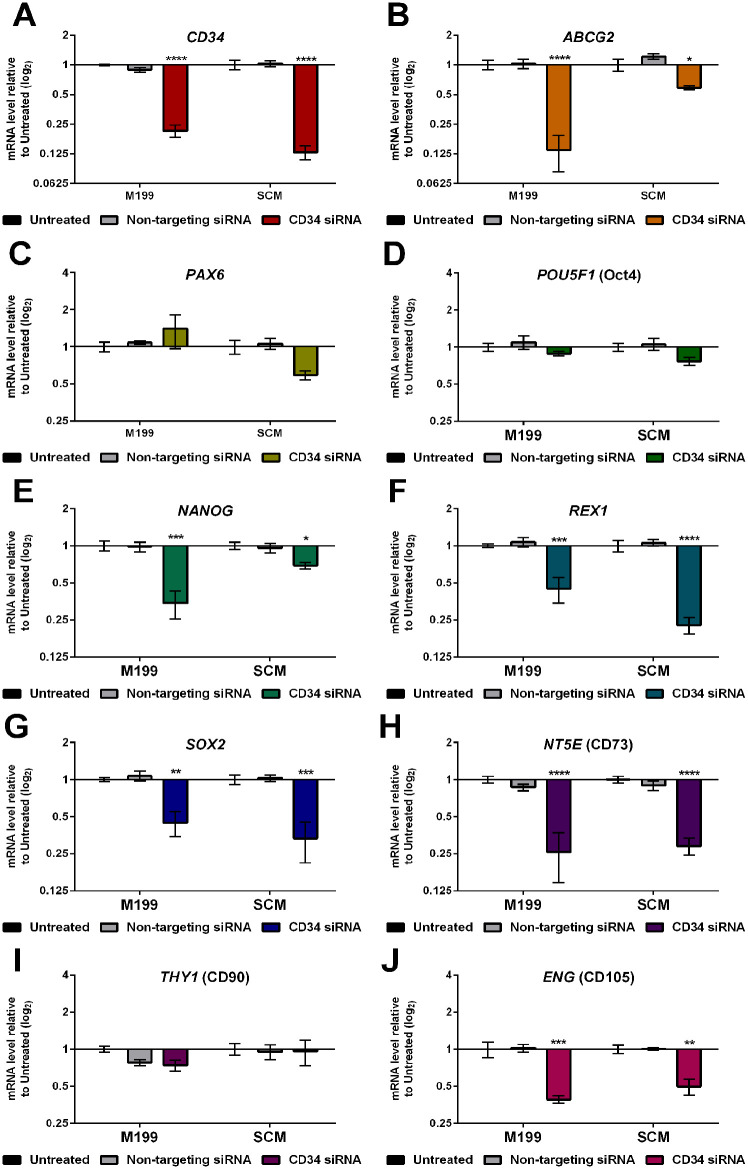
Effect of siRNA-mediated knockdown of CD34 on C-MSC gene expression. C-MSC cultured in either M199 or SCM were transfected with either non-targeting siRNA or CD34 siRNA at passage 2. Relative levels of mRNA following transfection were determined by RT-qPCR for the following genes: (A) *CD34* (B) *ABCG2*, (C) *PAX6*, (D) *POU5F1*, (E) *NANOG*, (F) *REX1*, (G) *SOX2*, (H) *NT5E*, (I) *THY1*, (J) *ENG*. Expression of each target gene was normalized to *GAPDH* and represented relative to the mRNA expression of untreated cells in the corresponding media. Data shown as mean ± SEM of three independent experiments with three different C-MSC donors (n = 3), each with two replicates. Statistical significance of knockdown vs. untreated cells: *P ≤ 0.05, **P ≤ 0.01, ***P ≤ 0.001, ****P ≤ 0.0001.

Knockdown of CD34 was achieved using siRNA transfection. The transfection process caused a maximum cell viability decrease of 30% (see Supplementary Fig. 2), unrelated to the CD34 knockdown and similar to the non-targeting siRNA control. The process achieved a knockdown efficiency of 78.6% ± 5.51% in M199 and 87.0% ± 3.65% in SCM (Fig. 6A). RT-qPCR was performed to assess the effect of knockdown of CD34 on other genes (Fig. 6). There was no significant effect on expression of any gene due to transfection with non-targeting siRNA. However, knockdown of CD34 caused a significant downregulation of *ABCG2* (Fig. 6B), *NANOG* (Fig. 6E), *REX1* (Fig. 6F), *SOX2* (Fig. 6G), *NT5E* (Fig. 6H), and *ENG* (Fig. 6J), in both M199 and SCM, suggesting that CD34 has some role in regulation of stem cell properties. Expression of *PAX6* (Fig. 6C), *POU5F1* (Fig. 6D), and *THY1* (Fig. 6I) were not significantly affected by CD34 knockdown.

### Response of C-MSC to Inflammatory Stimulus

**Figure 7.**
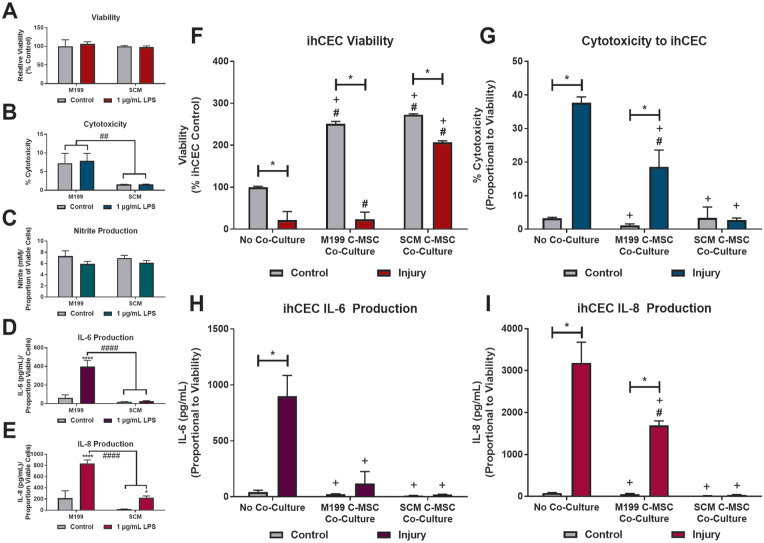
Potential anti-inflammatory effect of C-MSC cultured in SCM. (A-E) C-MSC cultured in M199 or SCM were exposed to 1 µg/mL LPS for 72 h. (A) Relative cell viability at 72 h. Data shown relative to control of same media. (B) Cytotoxic effect of LPS (LDH production). (C) Nitrite accumulation in the culture medium. (D) IL-6 production. (E) IL-8 accumulation in the culture medium. Data corrected for relative cell viability. B-E corrected for relative cell viability. Data shown as mean ± SEM of three independent experiment (n = 3) each with two replicates. Statistical significance of control vs. LPS: *P ≤ 0.05, ****P ≤ 0.0001. Statistical significance of M199 vs. SCM: ##*P* ≤ 0.01, ####*P* ≤ 0.0001. (F-I). Response of ihCEC injury model to co-culture with C-MSC. ihCEC treated with 20% ethanol for 30 s, followed by exposure to 1 µg/mL LPS. C-MSC, M199 or SCM cultured, were co-cultured with the ihCEC immediately after ethanol injury, during LPS stimulation. (F) Relative cell viability at 72 h. Data shown relative to no co-culture control. (G) Cytotoxic effect on ihCEC measured by LDH production. Data corrected for production of LDH by C-MSC-only controls and relative cell viability. (H) IL-6 accumulation in the culture medium. (D) IL-8 accumulation in the culture medium. G, H, I corrected for production by C-MSC-only controls and relative cell viability. All data shown as mean ± SEM of three independent experiment with three different C-MSC donors (n = 3) each with two replicates. Statistical significance of control vs. injury: *P ≤ 0.0001. Statistical significance vs. no co-culture control: #*P* ≤ 0.0001. Statistical significance vs. no co-culture injury: +*P* ≤ 0.0001.

Possible differences in response to inflammatory stimuli between cells cultured in M199 and SCM, were assessed by response to an LPS stimuli (Fig. 7). LPS had no effect on viability (Fig. 7A) or cytotoxicity (Fig. 7B) of cells in either media. Neither cell type produced nitric oxide, measured as nitrite, in response to the LPS stimulus (Fig. 7C). However, there was a difference in production of IL-6 (Fig. 7D) and IL-8 (Fig. 7E), in response to LPS. C-MSC that had been cultured in M199 showed significant production of both IL-6 and IL-8, compared to the control. C-MSC in SCM did not produce IL-6 in response to LPS but did produce IL-8; however, the amount produced was significantly lower than that produced by M199 C-MSC.

### Corneal Epithelial Cell Injury Model to Assess Anti-Inflammatory Potential of C-MSC

An injury model consisting of ihCEC treated with 20% ethanol for 30 s, followed by sustained exposure to 1 µg/mL LPS was used to assess anti-inflammatory potential of C-MSC cultured in M199 or SCM. C-MSC were co-cultured with the ihCEC, immediately after ethanol injury, during LPS stimulation. In the injury model without C-MSC co-culture, there was a significant drop in ihCEC viability at day 3, compared to the non-injured control (Fig. 7F). When co-cultured, the presence of the C-MSC caused increased ihCEC proliferation and in controls, ihCEC cell numbers were over double that of the no co-culture control. However, there was a significant drop in cell viability in injured co-cultures compared to associated control. In M199, this drop was substantial, with similar final viability to the no co-culture injury. When the injury model was cultured with SCM-C-MSC, the final ihCEC viability, although significant compared to the co-culture control, was significantly higher that the no co-culture control, suggesting that the SCM-C-MSC had a protective or proliferative effect on the injured cells. Injury had a significant cytotoxic effect on the ihCEC (Fig. 7G). This effect was also seen when injured ihCEC were cultured with M199-C-MSC, although the effect was significantly lower than the ihCEC alone. When co-cultured with SCM C-MSC there was no significant cytotoxic effect seen due to the injury. Due to injury, ihCEC released significant amounts of IL-6 (Fig. 7H) and IL-8 (Fig. 7I) into the medium, compared to the control. The production of IL-6 was inhibited in both the M199 and SCM co-cultures. The M199-C-MSC co-culture caused some reduction in IL-8 production, but the SCM-C-MSC co-culture caused a complete reduction. Injury to the ihCEC did not produce any significant nitric oxide production, regardless of co-culture (data not shown).

### Effect of C-MSC-CM on Proliferation, Wound Healing and (Lymph)Angiogenic Network Formation by LECs and BECs

**Figure 8.**
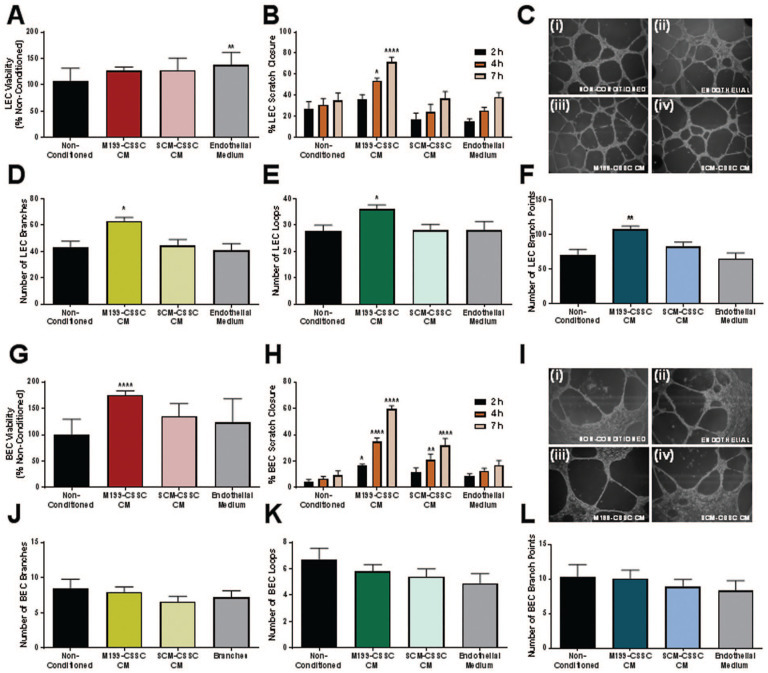
Effect of C-MSC CM on LEC and BEC. Effect of M199-CSS and SCM-C-MSC CM on proliferation of (A) LEC and (G) BEC was assessed after 24 h. Data shown relative to the non-conditioned control. Data shown as mean ± SEM of five independent experiments with conditioned medium from five different C-MSC donors each with five replicates. Statistical significance vs. non-conditioned control: *P ≤ 0.05, **P ≤ 0.01, ***P ≤ 0.001, ****P ≤ 0.0001. Effect on wound healing of (B) LEC and (H) BEC was assessed by scratch wound assay. Area of wound was measured at 2, 4, and 7 h. Data shown as mean ± SEM of five independent experiments each with n = 5. Statistical significance vs. non-conditioned control: *P ≤ 0.05, **P ≤ 0.01, ****P ≤ 0.0001. Effect of C-MSC CM on (lymph) angiogenic network formation of (C) LEC and (I) BEC using matrigel assays. Representative images shown of (i) non-conditioned medium, (ii) endothelial medium, (iii) M199-C-MSC CM, and (iv) SCM-C-MSC CM. Images were analyzed and number of branches (D, J), loops (E, K) and branch points (F, L) counted. Data shown as mean ± SD (n ≥ 11). Statistical significance vs. non-conditioned control: *P ≤ 0.05, **P ≤ 0.01.

Conditioned media from both M199 C-MSC and SCM C-MSC had a slight proliferative effect on LECs compared to the non-conditioned control (Fig. 8A). However, this was not as large an effect as having the cells in their normal endothelial growth medium. Results of the LEC scratch wound assay (Fig. 8B) showed that scratches cultured in M199-C-MSC CM closed significantly faster than in any other medium and SCM-C-MSC CM had no positive or negative effect on scratch closure. Tube network formation assays with LECs (Fig. 8C), showed that networks formed in M199-C-MSC CM had more branches (Fig. 8D), formed more loops (Fig. 8E) and had a larger number of branch points (Fig. 8F) than in all other media, effectively forming a more complex lymphangiogenic network. Culture in SCM-C-MSC CM had no positive or negative effect on network complexity of LEC.

M199-C-MSC CM had a proliferative effect on BECs, compared to non-conditioned medium and endothelial growth medium (Fig. 8G), and SCM-C-MSC CM did not have this effect. Results of the BEC scratch wound assay (Fig. 8H) showed that scratches cultured in M199-C-MSC CM and SCM-C-MSC CM closed significantly faster than in control medium, although the effect was more prominent in M199-C-MSC CM. Tube network formation assays (Fig. 8I) showed that BEC networks were similar in all media types and culture in C-MSC CM had no positive or negative effect on angiogenic network complexity.

## Discussion

In the last few decades, the study of MSCs as novel therapies for inflammatory and immune diseases has increased significantly and the discovery and characterization of MSCs derived from the cornea offers potential new treatment pathways for ocular surface disorders^5–8^. In developing therapies that incorporate C-MSC, it is important to ensure the optimal culture medium and environment is implemented for this cell type to get the ideal therapeutic response for the clinical need. Culture medium constituents can have a significant influence on cell behavior, differentiation and final secretome, all important aspects of any cell therapy.

The C-MSC phenotype is distinct from that of the *in vivo* keratocyte from which it is derived^5^, and it is currently not known whether this phenotype appears in a subset of cells within the cornea *in vivo* or if phenotypic change occurs after removing the cells from their 3D environment and culturing in 2D, with forced proliferative signals from serum or growth factors. However, C-MSC and corneal stromal cells have been shown to have beneficial therapeutic properties both *in vitro* and *in vivo*^12,13,22,28–30^ and optimizing the *in vitro* environment in which they are cultured could induce positive changes in the cell population that would make a more successful cell therapy.

In this study, we performed a further comparison of two different media that have both previously been shown to support a typical MSC phenotype when used in culture of C-MSC, as shown by expression of typical MSC markers CD73, CD90 and CD105, alongside differentiation down the mesenchymal lineage^7,11,12,24^. The majority of investigations into phenotype of MSC extracted from the cornea, have focused on the production of an MSC phenotype^7,18,31,32^, predominantly according to the now outdated minimal ISCT criteria^33^. Culture expanded MSCs, including C-MSC, have been shown to consist mostly of a heterogeneous population of cells exhibiting a spectrum of phenotypes and functional properties^34,35^ and the properties and phenotypes can be affected by the tissue, donor, species, isolation technique, culture protocols including medium, and number of prior cell doublings^4^.

The major difference between the two media in this study is that M199 contains a large percentage of undefined and animal-origin FBS, whereas SCM contains knockout serum-replacement, a chemically defined substitute^36^. Although both M199 and SCM supported expression of the MSC markers CD73, CD90 and CD105 homogenously across the population, there were definitive differences in the other proteins and genes that were expressed.

The SCM medium also contained a source of non-essential amino acids and the recombinant proteins bFGF and hLIF, that may have also contributed to the differing phenotype of the cultured C-MSC. The growth factor bFGF has been associated with maintenance of a keratocyte phenotype within the cornea and is also essential in maintaining pluripotency of hESC^37,38^. LIF is a protein that is essential in maintaining pluripotency in mouse ESC but has also been implicated in naïve hESC^39,40^.

During this study we focused on the ability of the medium to affect expression of the marker CD34, and the effect of selecting and knocking-down CD34 on the entire cell population. CD34 is a characteristic marker for quiescent keratocytes *in vivo*^41,42^ and has been linked to a number of other progenitor cell types^43^. It has been speculated that CD34 plays roles in regulation of differentiation, adhesion and quiescence^43^. However, it is usually accepted that MSC, no matter from where they are derived, should not express the marker CD34. Our group has previously disputed this fact^7,12,43^, hypothesizing that C-MSC lose CD34 protein expression during *in vitro* culture, but are always capable of producing it in the correct environment. By investigating RNA expression rather than protein expression we show that there is *CD34* gene expression in C-MSC expanded in both media; however, this is at a relatively higher level in SCM and is maintained more effectively across passage. This is also reflected in a higher level of protein expression as shown by immunocytochemistry.

When we sorted for cell populations that were actively expressing CD34 protein on the cell surface, no differences were seen in the CD34^+^ and CD34^−^ cell populations in terms of expression of MSC markers CD73, CD90, and CD105. This suggests that the expression of CD34 is independent of these constitutively expressed markers, and therefore expression of these markers is not dependent on culture medium. In M199, isolated cells actively expressing CD34 protein show increased gene expression of other pluripotency and progenitor markers. However, this effect is not seen when C-MSC are cultured in SCM, where expression levels of these markers are always significantly higher in both the CD34^−^ and CD34^+^ populations than in M199. This may indicate that the cells are more homogeneous population when cultured in SCM, and the expression of CD34 is of little consequence to the overall phenotype. In both media, *CD34* gene expression does not disappear in the CD34^−^ population, which may indicate that C-MSC retain the ability to produce the CD34 protein under certain conditions. The translation of the CD34 protein has been shown to be dependent on the methylation state of the *CD34* gene^44^, it has also been shown in hematopoietic stem cell culture that CD34^−^ and CD34^+^ cell populations are freely interconvertible^45^. When the *CD34* gene was knocked down or silenced, rather than selecting for the protein, the expression of pluripotency genes was also negatively affected, regardless of the culture medium. This demonstrates that although the CD34 protein may not be expressed in large amounts it is important for overall phenotype that the gene is able to be expressed. This indicates that the CD34 protein is expressed transiently depending on environment as has been previously evidenced in hematopoietic progenitors^44–46^. Using a culture medium that maintains more potential to express CD34 may result in a more homogeneous cell population with a better therapeutic potential *in vivo*.

This study intended to expand the gene markers investigated to cover transcription factors normally associated with pluripotent stem cells. Although the role of these transcription factors is well described in embryonic stem cells, their expression and role in MSCs is not as well-defined and can still be considered controversial^47^. To our knowledge, this is the first time the gene expression of *NANOG*, *REX1*, and *SOX2* has been reported in C-MSC. These three markers along with *CD34*, *ABCG2*, *PAX6*, and *CD90* were all expressed at relatively higher levels in SCM than in M199. The expression of *OCT4A*, *SOX2*, *NANOG* and *REX1* are all associated with one another and all are associated with self-renewal and pluripotency of stem cells^48^. They have all been previously described as being expressed by various types of MSC including bone marrow, umbilical cord, dermal and cardiac MSC^49–52^. The expression of these transcription factors indicates increased life-span and self-renewal in MSCs^53^; therefore, the increased expression of these markers when C-MSC are cultured in SCM indicates the ability to culture for longer periods of time without adverse effects.

The use of MSCs as cell therapies to modulate the innate and adaptive immune systems^54^ suggests C-MSC have the potential to be developed as a treatment for inflammatory disorders of the ocular surface, eliciting a wound healing response through paracrine signaling^55^. Due to the fact that systemic delivery often results in stem cells getting caught in the pulmonary passages^56^, we believe that it will be more efficient to apply C-MSC topically to an injured and inflamed ocular surface, where they can assert their paracrine healing effects through the tear film, directly to the corneal epithelial cells^2^.

To investigate the paracrine healing effects of C-MSC an *in vitro* model of corneal epithelial inflammation was developed previously that begins with an initial injury of 20% (v/v) ethanol applied to corneal epithelial cells for 30 s followed by stimulation with interleukin-1β in the culture medium to mimic inflammation^6^. When subsequently co-cultured with our C-MSC this previous study demonstrated a potent anti-inflammatory potential of the stem cells. This study simulated the injury in a different manner; first damaging the corneal epithelial cells with ethanol but then applying an LPS stimulus to mimic an infection such as bacterial keratitis. Bacterial LPS is a component involved in triggering the inflammatory process in human cells by interacting with toll-like receptor 4 and stimulating an intracellular inflammatory cascade^57^ that includes production of the proinflammatory cytokines IL-6 and IL-8^58,59^. C-MSC, particularly when cultured in SCM do not respond to the presence of LPS by losing significant viability or cell lysis. They also do not produce nitrite, an indicator of nitric oxide released through the inducible nitric oxide synthase pathway indicated during inflammation. LPS stimulation of C-MSCs does lead to increased production of IL-6 in M199 but not in SCM and increased levels IL-8 in both media; however, the levels were about approximately 10 times lower than that produced by corneal epithelial cells in response to LPS, indicating a diminished response to bacterial infection. When C-MSC were co-cultured with the corneal epithelial cell injury model, a reduction in the levels of inflammatory cytokines was seen, along with an increase in the number of cells surviving. This anti-inflammatory response was seen to a higher level with C-MSCs that had been cultured in SCM.

In many cell therapy applications for other tissues and organs there is a wish for MSCs to have a positive angiogenic effect, and there is evidence that this can be affected by the paracrine secretions of MSCs^60^. However, the absence of blood vessels in the cornea is integral to its function as a transparent tissue^61^. Therefore, an angiogenic response to a C-MSC therapy for the cornea would potentially be harmful. In this study, M199-C-MSC appeared to promote LEC and BEC migration and tube formation, whereas SCM-C-MSC tended to a more anti-angiogenic phenotype, which would be important for maintaining an avascular cornea, adding more weight to the argument that SCM produces an MSC with a more beneficial phenotype for a cell therapy.

In this study we have shown that a simple change in the culture medium of a cell type can lead to significant changes in protein and gene expression of the cells. In this case, it led to a beneficial change; however, other changes in environment may be detrimental, and the consequences of altering the phenotype of a cell therapy must be explored in detail, further than we have managed to provide in this manuscript.

Scalability is vital for cell therapies, particularly due to the disparity between the very low abundance of cells initially isolated, compared to the quantity required for a cellular product. SCM, as used in this study, is a more complex and expensive culture medium than M199, so would require a much higher outlay if the product was to be scaled-up. However, SCM has much more scope for clinical use as it does not contain any ingredients of animal origin and GMP-version of all components are already available. To produce the required number of cells for a cell therapy there would potentially have to be extended passage of the cells and it would be vital that throughout the process of cell manufacture, monitoring, and characterization takes place, to guarantee minimal change to cell properties and potency. A further study looking at the stability of protein and gene expression over extended passages in our optimized media is therefore required in future.

In conclusion, the use of an optimized culture medium, such as SCM, may be key in creating stem cell populations that are more suitable for certain therapeutic targets. Our eventual aim is to develop a C-MSC-based therapy that can be applied topically to the ocular surface^2^. For this purpose, culture in a medium similar to SCM would be more suitable due to increased anti-inflammatory potential when exposed to injured corneal epithelial cells and decreased probability of angiogenesis when compared to culture in a serum-based medium.

## Supplemental Material

sj-docx-1-cll-10.1177_09636897241241992 – Supplemental material for Increased Anti-Inflammatory Therapeutic Potential and Progenitor Marker Expression of Corneal Mesenchymal Stem Cells Cultured in an Optimized Propagation Medium Supplemental material, sj-docx-1-cll-10.1177_09636897241241992 for Increased Anti-Inflammatory Therapeutic Potential and Progenitor Marker Expression of Corneal Mesenchymal Stem Cells Cultured in an Optimized Propagation Medium by Andrew Hopkinson, Maria Notara, Claus Cursiefen and Laura E. Sidney in Cell Transplantation

sj-docx-2-cll-10.1177_09636897241241992 – Supplemental material for Increased Anti-Inflammatory Therapeutic Potential and Progenitor Marker Expression of Corneal Mesenchymal Stem Cells Cultured in an Optimized Propagation Medium Supplemental material, sj-docx-2-cll-10.1177_09636897241241992 for Increased Anti-Inflammatory Therapeutic Potential and Progenitor Marker Expression of Corneal Mesenchymal Stem Cells Cultured in an Optimized Propagation Medium by Andrew Hopkinson, Maria Notara, Claus Cursiefen and Laura E. Sidney in Cell Transplantation
