# Supplementary material for: Increased Anti-Inflammatory Therapeutic Potential and Progenitor Marker Expression of Corneal Mesenchymal Stem Cells Cultured in an Optimized Propagation Medium
Source: Cell Transplant. 2024 Apr 11;33:09636897241241992. doi: 10.1177/09636897241241992 (PMC11010753; doi:10.1177/09636897241241992)
Supplement: sj-docx-1-cll-10.1177_09636897241241992 – Supplemental material for Increased Anti-Inflammatory Therapeutic Potential and Progenitor Marker Expression of Corneal Mesenchymal Stem Cells Cultured in an Optimized Propagation Medium [file sj-docx-1-cll-10.1177_09636897241241992.docx]

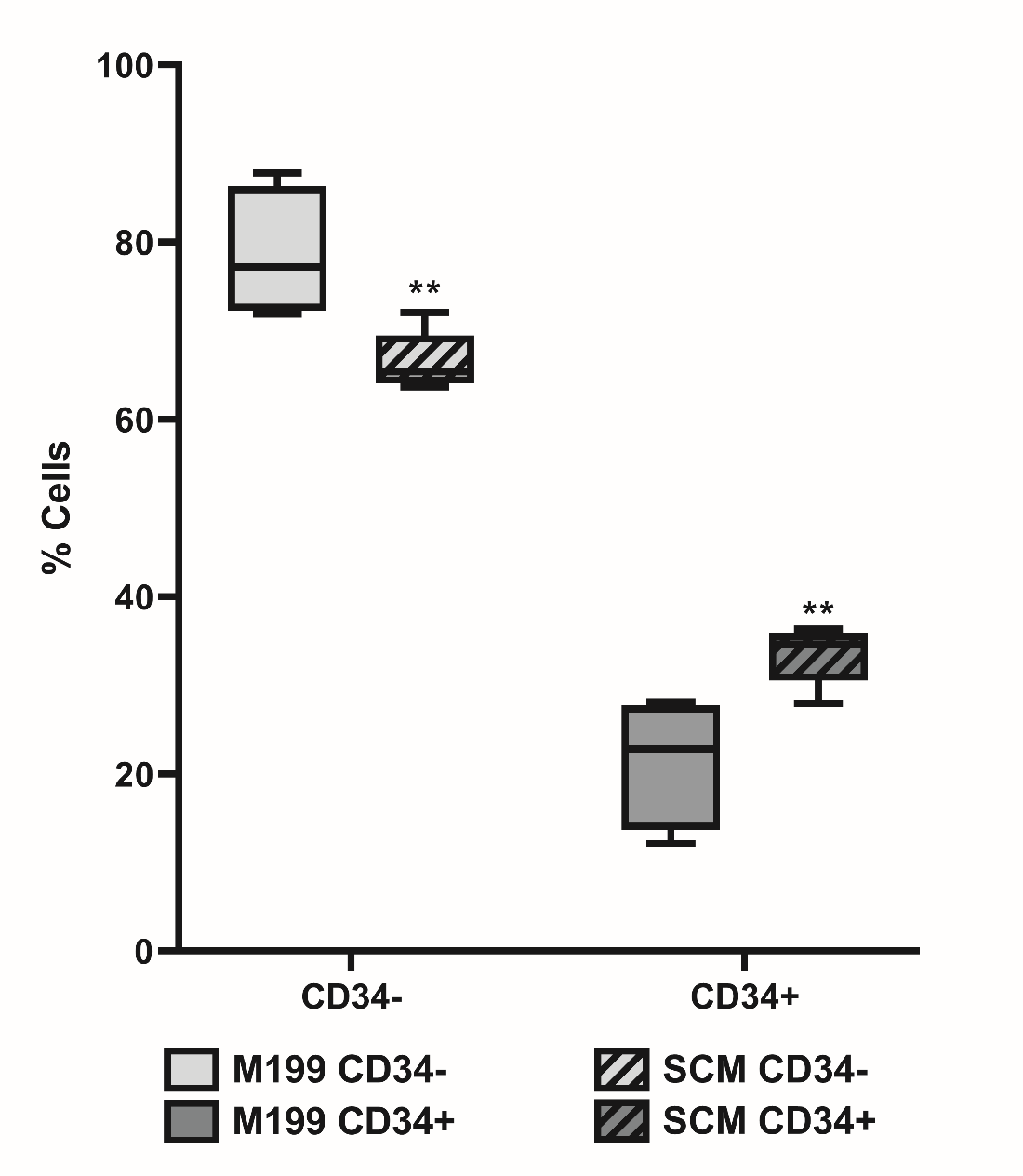


**Supplemental Figure 1. Percentage of C-MSC expressing CD34.** C-MSC were cultured for one passage in either M199 or SCM. Cell sorting for positive CD34 expression was performed by MACS and number of cells in each population counted**.** Data shown as boxplot of 5 independent experiments (5 donors, n=5). Statistical significance SCM vs. M199 : **p≤0.01.


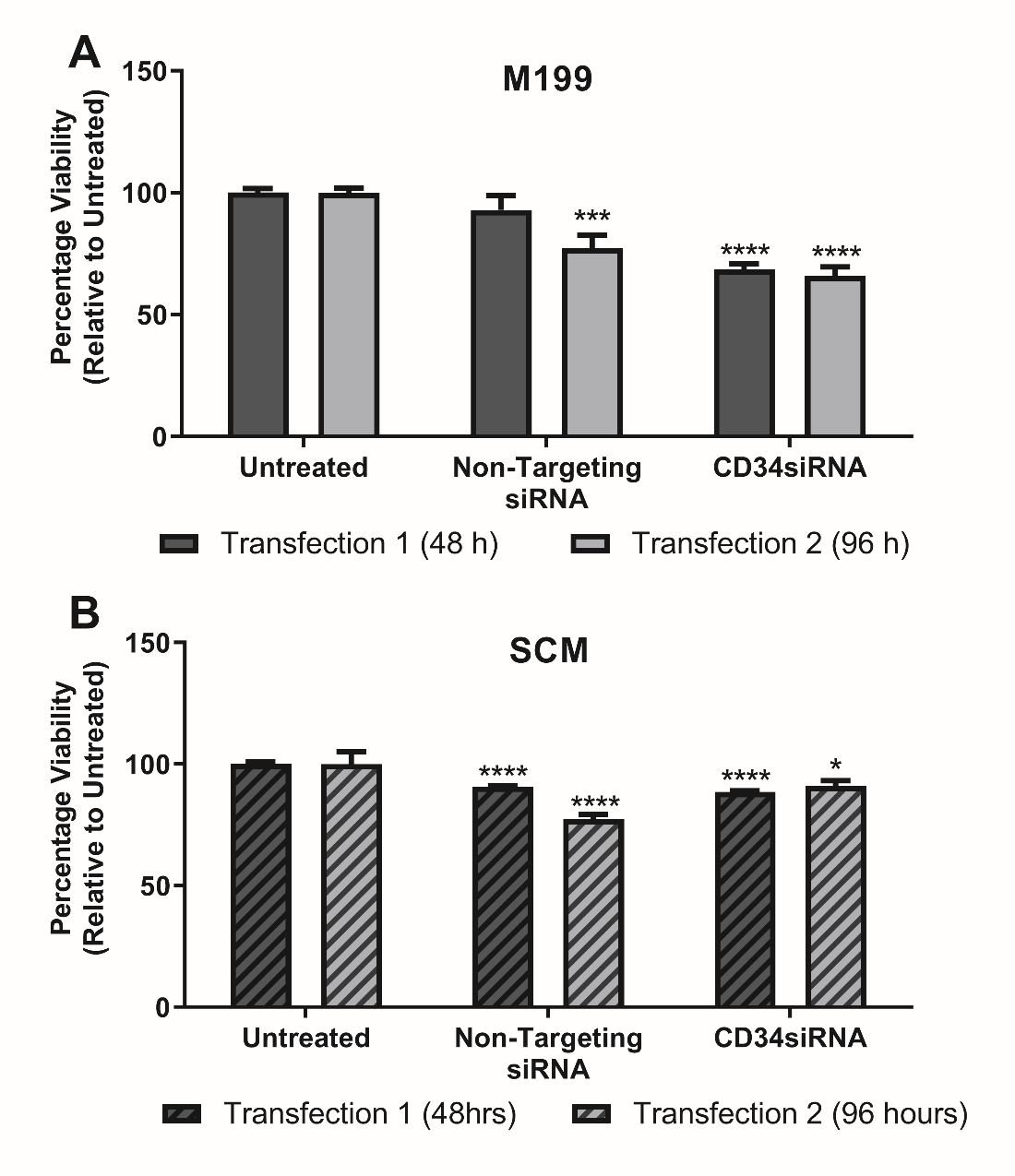


**Supplemental Figure 2. Effect of siRNA-mediated knockdown on viability.** C-MSC cultured in either (A) M199 or (B) SCM were transfected with either non-targeting siRNA or CD34 siRNA at passage 2. Two transfections were performed, with the second transfection at 48 hours after the first. Cell viability was assessed 48 hours after each transfection. Data is shown relative to viability of untreated C-MSC at the corresponding timepoint. Data shown as mean±SEM of 3 independent experiments (n=3), each with 3 replicates. Statistical significance of knockdown vs. untreated cells: *p≤0.05, ***p≤0.001, ****p≤0.0001.
