# Supplementary material for: Increased Anti-Inflammatory Therapeutic Potential and Progenitor Marker Expression of Corneal Mesenchymal Stem Cells Cultured in an Optimized Propagation Medium
Source: Cell Transplant. 2024 Apr 11;33:09636897241241992. doi: 10.1177/09636897241241992 (PMC11010753; doi:10.1177/09636897241241992)
Supplement: sj-docx-2-cll-10.1177_09636897241241992 – Supplemental material for Increased Anti-Inflammatory Therapeutic Potential and Progenitor Marker Expression of Corneal Mesenchymal Stem Cells Cultured in an Optimized Propagation Medium [file sj-docx-2-cll-10.1177_09636897241241992.docx]

**Supplemental Table 1.** Primary antibody information for immunocytochemistry

| **Antigen** | **Clone** | **Source (Catalogue Number)** | **Host** |
| --- | --- | --- | --- |
| Vimentin | V9 | Vector Labs (VPV684) | Mouse |
| PanCytokeratin | C-11 | Thermo Scientific Pierce (MA1-19043) | Mouse |
| CD34 | 581 | Sigma-Aldrich (SAB4700736) | Mouse |
| CD105 | Polyclonal | R&D Systems (AF1097) | Goat |
| CD90 | F15-42-1 | Thermo Scientific Pierce (MA5-16671) | Mouse |
| CD73 | Polyclonal | Thermo Scientific Pierce (PA5-11871) | Rabbit |
| ABCG2 | 5D3 | R&D Systems (MAB995) | Mouse |
| SSEA-4 | MC813-70 | R&D Systems (MAB1435) | Mouse |
| Oct-4A | 653108 | R&D Systems (MAB17591) | Mouse |
| α-SMA | 1A4 | Abcam (ab7817) | Mouse |
| ALDH3A1 | Polyclonal | Abcam (ab76976) | Rabbit |
| Keratocan | Polyclonal | Santa Cruz Biotechnology (sc-33243) | Goat |

**Supplemental Table 2.** Taqman Probe Information

| **Gene Name** | **Protein Name** | **Assay ID** |
| --- | --- | --- |
| *GAPDH* | Glyceraldehyde 3-phosphate dehydrogenase | Hs99999905_m1 |
| *ABCG2* | ATP-binding cassette sub-family G member 2 | Hs01053790_m1 |
| *CD34* | CD34 | Hs00990732_m1 |
| *ENG* | CD105 (Endoglin) | Hs00923996_m1 |
| *NANOG* | Nanog | Hs04260366_g1 |
| *NT5E* | CD73 (Ecto-5’-nucleotidase) | Hs01573922_m1 |
| *PAX6* | Paired box 6 | Hs00240871_m1 |
| *POU5F1* | POU domain, class 5, transcription factor 1 (Oct4) | Hs00999634_gH |
| *SOX2* | SRY (sex determining region Y)-box 2 | Hs01053049_s1 |
| *THY1* | CD90 (Thymocyte differentiation antigen 1) | Hs00174816_m1 |

**Supplemental Table 3.** Conjugated antibody information for flow cytometry

| **Antigen** | **Clone** | **Source (Catalogue Number)** | **Fluorophore** |
| --- | --- | --- | --- |
| CD34 | 4H11 | Affymetrix eBioscience (11-0349-42) | FITC |
| CD73 | AD2 | Affymetrix eBioscience (12-0739-42) | PE |
| CD90 | 5E10 | Affymetrix eBioscience (15-0909-42) | PE-Cy5 |
| CD105 | SN6 | Affymetrix eBioscience (12-1057-42) | PE |
| SSEA-4 | MC0813-70 | Affymetrix eBioscience (12-8843-42) | PE |
| ABCG2 | 5D3 | Affymetrix eBioscience (12-8888-42) | PE |
